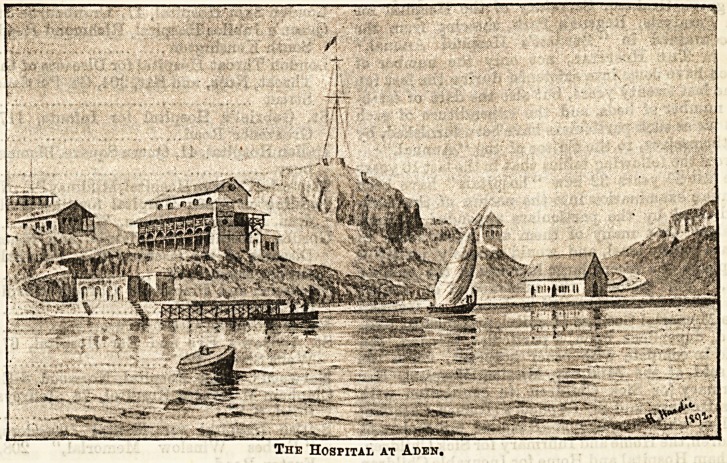# The New Hospital, Aden

**Published:** 1892-07-23

**Authors:** 


### HOSPITAL CONSTRUCTION.

### THE NEW HOSPITAL AT ADEN.

At most ports all over the world, and, indeed, wherever an English colony is formed, the establishment of a hospital is inevitable. The latest addition to the hospital ranks abroad is the New General European Hospital at Aden, of which, through the kindness of the proprietors of the *Graphic*, we give an illustration. The little institution, which was opened in May, is a very quaint-looking building, standing high above the sea, and commanding a lovely view. It was opened with much ceremony by General Jopp, the Military Governor and Resident. A large gathering assembled on the occasion, which included many military and naval officers and native grandees. Previous to the erection of the new hospital the accommodation was both insufficient and unsuitable, especially in respect to ventilation. The new hospital is both commodious and airy. The first storey in the main building has been reserved for naval patients: 20,000 rupees of the total 120,000 rupees expended in the erection of the building having been subscribed by members of the Navy. The hospital contains separate wards for women and children, observation, and mental cases, and is thus well fitted to meet all that is required of it.

THE HOSPITAL AT ADEN.

## Figures and Tables

**Figure f1:**